# Habitat Management: A Tool to Modify Ecosystem Impacts of Nitrogen Deposition?

**DOI:** 10.1100/tsw.2001.379

**Published:** 2001-12-05

**Authors:** S.A. Power, C.G. Barker, E.A. Allchin, M.R. Ashmore, J.N.B. Bell

**Affiliations:** Department of Environmental Science and Technology, Imperial College, Ascot, Berkshire, UK

**Keywords:** nitrogen deposition, habitat management, heathland, Calluna, ecosystem recovery, burning, mowing

## Abstract

Atmospheric nitrogen deposition has been shown to affect both the structure and the function of heathland ecosystems. Heathlands are semi-natural habitats and, as such, undergo regular management by mowing or burning. Different forms of management remove more or less nutrients from the system, so habitat management has the potential to mitigate some of the effects of atmospheric deposition. Data from a dynamic vegetation model and two field experiments are presented. The first involves nitrogen addition following different forms of habitat management. The second tests the use of habitat management to promote heathland recovery after a reduction in nitrogen deposition. Both modelling and experimental approaches suggest that plant and microbial response to nitrogen is affected by management. Shoot growth and rates of decomposition were lowest in plots managed using more intensive techniques, including mowing with litter removal and a high temperature burn. Field data also indicate that ecosystem recovery from prolonged elevated inputs of nitrogen may take many years, or even decades, even after the removal of plant and litter nitrogen stores which accompanies the more intensive forms of habitat management.

## INTRODUCTION

There is now strong evidence that anthropogenic nitrogen deposition has contributed to widespread changes in both the structure and function of many ecosystems throughout Europe and North America[1,2,3,4,5]. European heathlands, dominated by evergreen *Ericacae*, have been in decline throughout the past century, with less than 10% of their former extent currently remaining[6,7]. The recent loss of *Calluna* dominance in areas receiving high deposition inputs of nitrogen, and results from studies in both The Netherlands and the U.K., indicate that atmospheric nitrogen has played a pivotal role in heathland decline over the past few decades. A significant relationship between nitrogen deposition and foliar chemistry of *Calluna* at heathland sites has been demonstrated in field surveys and experiments[8,9,10,11]. Furthermore, increased production of plant biomass and (nitrogen enriched[11]) litter in response to nitrogen addition results in an accumulation of soil organic nitrogen stores[11]. This build up of nitrogen within the system, in combination with an accelerated decomposition rate of *Calluna* litter in response to nitrogen addition[12,13,14], can thus lead to a significant increase in the rate of nutrient turnover in heathland systems. Litter and soil organic pools therefore represent a substantial store of nitrogen, the release of which can both exacerbate and prolong the effects of atmospheric deposition inputs.

Heathlands are semi-natural ecosystems undergoing regular management removal of biomass, and thus nutrients, in order to maintain a low nutrient environment. For lowland heathlands (i.e., <300 m altitude), typical managements include mowing, burning, grazing, and turf cutting[15]. The type and frequency of habitat management determines the amount of nitrogen removed in plant, litter, and humus compartments. Habitat management therefore has a major impact on the nitrogen status of heathland ecosystems and, by reducing nitrogen stores, has the potential to affect ecosystem response to atmospheric inputs of nitrogen.

The objectives of the study described in this paper were (1) to evaluate the effectiveness of different management regimes at reducing the impact of ongoing inputs of reactive nitrogen; (2) to assess the role of habitat management as a tool to accelerate ecosystem recovery following historical elevated inputs of nitrogen; and (3) to investigate interactions between habitat management and nitrogen deposition on heathland systems over realistic time scales, using a modelling approach.

## METHODS

### The Field Site

Field experiments were carried out at a lowland heathland in Surrey, England, within the Thursley Common National Nature Reserve. Two related experiments are described: (1) the new (management-nitrogen) study and (2) the recovery study. The new study involves ongoing nitrogen additions following four types of habitat management that took place in February 1998. The recovery study, on the other hand, used plots that had previously received 7 years of nitrogen additions (1989 to 1996) followed by management in February 1998, but with no further nitrogen additions after 1996. Background deposition at the site has been estimated at 10 to 15 kg N ha$^{-1}$ year$^{-1}$, just below the critical load of 15 to 20 kg N ha$^{-1}$ year$^{-1}$, for lowland heathlands[2]. Further site details can be found in references 11, 13, 16, 17, and 18.

### *The Management-Nitrogen Study*

Four experimental blocks, each containing two 4- × 4-m plots were established in January 1998. Each plot was subdivided into four subplots, to which one of the following four managements was carried out:

- Low intensity mow: above-ground biomass cut at 15-cm height using a power scythe, and cut vegetation removed. The litter layer remained intact.
- High intensity mow: *Calluna* plants cut at ground level and the litter layer removed, using a tractor driven forage harvester.
- Management (low temperature) burn: subplots ignited and all above-ground vegetation burnt. The litter layer remained untouched by the fire.
- Simulated accidental burn: although not a management as such, accidental summer fires are a frequent occurrence on lowland heathlands in the U.K. As with turf cutting, high temperature summer fires remove almost the entire litter and humus layers. This burn was carried out in the same way as the management burn with litter and humus layers being additionally dried, ignited, and left to smolder for 10 h. The remaining ash was then returned to the original subplots.

Nitrogen was added to the plots, every 2 weeks, in the form of a fine ammonium sulfate mist. Two levels of nitrogen addition were used (0 and 30 kg ha$^{-1}$ year$^{-1}$). Both control and nitrogen addition plots received 15 l of artificial rain solution (based on concentrations of cations and anions recorded in rainfall at a nearby site[17]) on each spraying visit. The concentration of ammonium ions applied to the nitrogen addition plots was 5.39 meq l$^{-1}$.

### *The Recovery Study*

A previous experiment between 1989 and 1996 involving the addition of nitrogen at a rate of 0 (control), 7.7 (low), or 15.4 (high) kg ha$^{-1}$ year$^{-1}$ was used as the basis for an investigation into the role of management in promoting ecosystem recovery following a reduction in nitrogen inputs. A full description of ecosystem responses to earlier nitrogen additions has been published elsewhere[11,16,17,18]. The same management techniques as described above were applied to four replicates of each of the three nitrogen treatment plots in February 1998, 17 months after the cessation of experimental nitrogen additions. Only data from the control and high nitrogen plots are presented in this paper.

#### *Above-Ground Responses*

Shoot length and percent *Calluna* cover were measured at the end of the growing season at 25 evenly distributed points per subplot (October 1998, October 1999, and November 2000). Seedling establishment (numbers of each species per subplot) was recorded in September 1999.

#### *Below-Ground Responses*

Post-management total soil nitrogen contents were determined in June 1998 using soil cores to a depth of 10 cm. Samples were digested using a sulphuric acid/selenium mixture[19] and total nitrogen content was determined using a Kjeltech nitrogen analyser. As a result of missing data, results are not presented for the low intensity mow treatment. Rates of litter decomposition were assessed using litter bags constructed from nylon mesh with a diameter of 0.5 mm. Litter was collected from each of the low mow, management burn, and high mow plots after management had been carried out; no litter remained in the simulated accidental fire plots, so this treatment was excluded from the decomposition study. Litter was replaced, in 10 replicate mesh bags per subplot, into plots from which it originated and two samples were collected every 12 weeks, oven dried, and weighed .

### Modelling

A simulation model, HeathN, was constructed in the Stella software package (High Performance Systems, U.S.) to simulate the growth and nitrogen dynamics of *C. vulgaris* under different rates of atmospheric nitrogen deposition. HeathN was developed in order to address some of the limitations of an existing Dutch model of heathland response to nitrogen[20]. The most significant differences between the Dutch modelling approach and HeathN include parameterisation with U.K. growth data to provide realistic simulations of biomass accumulation for British heathlands and the separation of biomass and foliar nitrogen concentration responses to deposition inputs. Growth data for *Calluna,* and its response to nitrogen, were obtained from the published literature[8,11,13,16,21,22,23,24] and unpublished results from heathland manipulation experiments in the U.K. (C. Barker, S. Power, S. Caporn, L. Cawley, M. Pilkington, and D. Wilson, personal communication). In the model, annual biomass, production is split into woody and green material. Each plant compartment contributes litter to the litter layer, which then decomposes at a constant rate of 20% per year[25]. A lowland parameterisation using growth and nitrogen data from lowland heathland systems has been used for the simulation runs presented in the results section below. Model validation was carried out by comparison with further data sets from the published literature. Model outputs for biomass accumulation and partitioning were found to closely reflect field data.

Heather beetle outbreaks occur stochastically, with a modelled probability, above thresholds of both *Calluna* and litter biomass, following a similar procedure to that used in the Dutch model of *Calluna* and *Deschampsia* competition[20]. One fundamental difference between these two modelling approaches is, however, that in HeathN, the probability of an outbreak increases with increasing foliar nitrogen concentrations. Following an outbreak, *Calluna* biomass is reduced by 95%. Because of the stochastic nature of beetle outbreaks, data are presented as means of 50 model runs.

Habitat management is simulated in the model by the removal of biomass (and nitrogen) from the system at user-defined intervals. Two types of management are simulated here: a low intensity mow with above-ground vegetation removed at a 15-year interval and a high intensity mow, which removes both above-ground and litter layers at the same interval. A full description of the model structure and parameterisation will be published shortly[26].

### Statistical Analysis

The effects of nitrogen and management treatments, and their interactions, were analysed using two-way ANOVA in Statistica. Post hoc comparisons were carried out on treatment means using the Scheffe test.

## RESULTS AND DISCUSSION

### Post-Management Nitrogen Addition

Several above-ground parameters were significantly stimulated by nitrogen addition following management. For example shoot length increased in response to nitrogen (Fig. 1). This response was statistically significant in both 1999 ($F_{1,24}$ = 38.0, $p < 0.001$) and 2000 ($F_{1,24}$ = 16.3, $p < 0.001$). Management also had a significant effect on shoot length in 1999 ($F_{3,24}$ = 20.4, $p < 0.001$); *Calluna* achieved greater growth in less intensively managed treatments (low mow and management burn) than in those which underwent more intensive managements (high mow and simulated accidental burn). Furthermore, there was also evidence that intensively managed plots responded (proportionally) more to atmospheric inputs of nitrogen, resulting in a significant interaction between nitrogen and management treatments in 1999 ($F_{3,24}$ = 3.26, $p < 0.05$). Nitrogen addition increased plant growth by an average of 39.2% in intensive managements in 1999. This compares to a mean increase of 15.0% in management burn and low mow plots. While the interaction was not statistically significant in 2000, the same trend was still seen. It appears that managements that remove a significant proportion of the nitrogen stored in the heathland system may be effective at reducing absolute plant growth responses to nitrogen. It is still too early, however, to know how long this effect will persist.

### Below-Ground

The rate of litter decomposition was significantly affected by both nitrogen addition ($F_{1,18\ (36\ weeks)}$ = 23.7, $p < 0.001$) and management ($F_{3,18\ (36\ weeks)}$ = 16.9, $p < 0.001$). Decomposition was fastest in those plots that received additional nitrogen and that had been managed by the low intensity mow. Fig. 2 illustrates the effect of nitrogen addition on the rate of weight loss of *Calluna* litter for low- and high-intensity mowing treatments. Although the interaction between nitrogen and management was not statistically significant, the proportional response to nitrogen addition

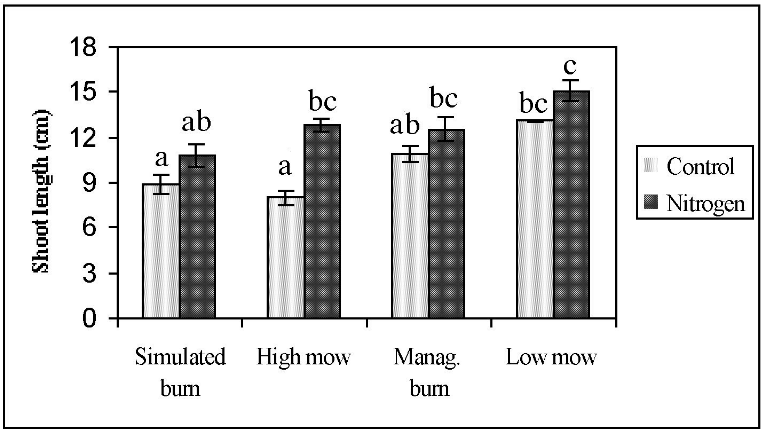

**FIGURE 1.** The effect of management and ongoing nitrogen addition on shoot growth of *Calluna* in 1999. *Note:* Bars represent ±1 standard error. Values with the same letters are not significantly different.

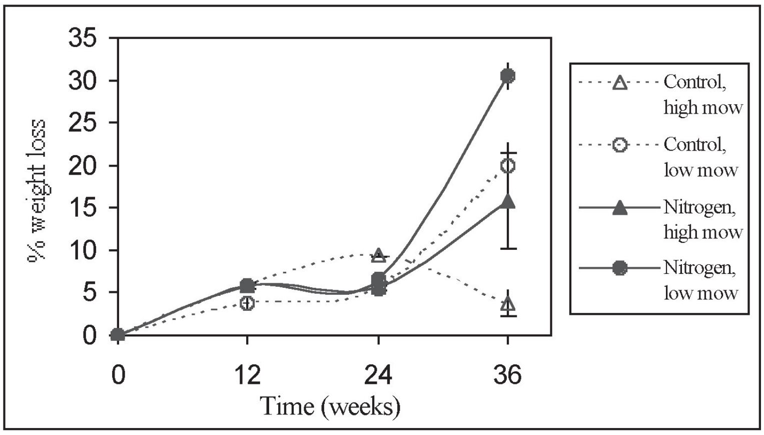

**FIGURE 2.** Litter decomposition in response to nitrogen addition. *Note:* Bars represent ±1 standard error.

was higher in the high intensity mow (+310%) than in the low mow (+53%). Since litter was collected before nitrogen additions began, the quality of the substrate being decomposed was uniform between treatments in this particular study. The effects of both nitrogen and management must therefore have occurred via the soil environment with either the activity or biomass (or both) of the decomposer microorganism community being stimulated by inorganic nitrogen addition. Earlier work at the same site, using cotton strips to assess the rate of cellulose decomposition[27], also indicated that microbial activity was stimulated in response to nitrogen addition[11]. The lower rate of decomposition in intensively managed plots suggests that the microbial population is significantly disturbed by this form of management and that the rate of nutrient turnover is reduced. More intensive managements thus appear to be more effective at maintaining a low nutrient environment, at least in the early years following litter/humus removal.

## Seedling Invasion

One of the key components of heathland decline, also used as an indicator of critical load exceedance[28], is a shift in dominance from *Calluna* to grasses. For dry heathlands, the grass involved is typically *Deschampsia flexuosa*. While initial invasion of grass seedlings following habitat management does not necessarily reflect long-term persistence, seedling establishment may provide some insight into the importance of nitrogen availability and gap creation in facilitating invasion by species other than dwarf shrubs.

Although seedling establishment was relatively low following management, there was a significant effect of both management and nitrogen addition on the number of *Deschampsia* seedlings established in treatment plots 16 months after nitrogen additions commenced (Table 1). The simulated accidental burn resulted in a large increase in the number of seedlings establishing compared to the other managements. These plots also had the greatest stimulation in response to nitrogen addition, resulting in a significant interaction between nitrogen and management.

These data suggest that the potential for natural invasion by grass seedlings may be increased by nitrogen deposition, particularly when activities such as accidental fires or perhaps turf cutting reduce vegetative regeneration from *Calluna* root stocks. The percentage cover of the *Calluna* canopy was significantly lower ($F_{3,24} = 5.1, p < 0.01$ in 1999, $F_{3,24} = 14.5, p < 0.05$ in 2000) following the simulated accidental burn (53.7% in 1999,

**TABLE 1**
**Effects of Habitat Management on Seedling Establishment by *Deschampsia***

| Management | Control (0 kg ha$^{-1}$ year$^{-1}$) | Nitrogen (30 kg ha$^{-1}$ year$^{-1}$) |
|---|---|---|
| Low intensity mow | 0.38 [a] | 1.00 [a] |
| Management burn | 1.12 [a] | 1.62 [a] |
| High intensity mow | 0.75 [a] | 1.50 [a] |
| Simulated accidental burn | 2.75 [a] | 6.88 [b] |
| | Nitrogen: $F_{1,24} = 15.0$, $p < 0.001$ | |
| | Management: $F_{3,24} = 24.0$, $p < 0.001$ | |
| | Interaction: $F_{3,24} = 5.1$, $p < 0.01$ | |

*Note:* Values represent mean number of seedlings per 2- × 2-m subplot. Means with different letters are significantly different.

69.0% in 2000) than after the low intensity mow (89.5% in 1999, 89.8% in 2000). It seems likely that the larger area of bare ground in the former plots provided conditions conducive to invasion by grass seedlings, particularly in plots receiving additional nitrogen. There thus appears to be a conflict in the initial post-management years between adequate nutrient removal and damage to *Calluna* root stocks, resulting in reduced regeneration. It remains to be seen, however, whether vigorous *Calluna* growth in the next few years will lead to the grasses being outcompeted.

While there have been many studies into the effects of either management or nitrogen addition on semi-natural ecosystems, very little work has so far looked at interactions between the two. One other published study indicates that management, in this case cutting, alters the response to chalk grasslands to nitrogen addition[29]. The current study therefore provides the first evidence that the form of management may influence plant and microbial response to atmospheric inputs of nitrogen.

## Ecosystem Recovery

The earlier manipulation experiment carried out at this field site (1989 to 1996) demonstrated that the majority of the nitrogen (approximately 75%) added over a 7-year period (totalling 99.8 kg $ha^{-1}$) was accumulated in the litter and soil layers. It is clear, therefore, that managements removing just above-ground plant material (i.e., the low intensity mow and management burn) thus remove only a small proportion of the nitrogen accumulated within a heathland system. The simulated accidental burn, or a turf cut, which removes almost all of the organic nitrogen stores might therefore be expected to accelerate the recovery of ecosystems exposed to elevated atmospheric inputs of nitrogen.

### *Below-Ground*

Management, to varying degrees, significantly reduced the amount of nitrogen within the heathland system. There was still some evidence of higher soil nitrogen contents in plots that had previously received nitrogen additions, although the effect of nitrogen was not significant after management had taken place (Table 2). The most intensively managed plots (simulated accidental burn) had lower levels of soil nitrogen than those where just the above-ground plant biomass was removed. However, there was no evidence that the simulated accidental burn was more effective than the other managements at reducing the difference in soil nitrogen contents between earlier control and nitrogen addition treatments.

The amount of nitrogen lost from vegetation during burning is dependent on fire temperature[30]. It is probable that the simulation of an accidental fire was carried out at a lower temperature than would be achieved in a real situation and that the remaining ash was therefore of a relatively high nitrogen content. In contrast, the removal of the entire humus layer in turf cutting would be expected to remove almost all of the organic nitrogen stored within the system[31], thus negating any residual treatment effects on nitrogen stores.

Despite the fact that the litter nitrogen concentrations were significantly higher ($F_{2,9} = 5.51$, $p < 0.05$) in plots that had previously received nitrogen additions (1.0% dry weight) compared to control plots (0.82% dry weight), no significant effect of earlier N treatment was found on the rate of litter decomposition following management. Decomposition rates were also not significantly different between managements, although litter in the low mow plots decomposed faster (23% mass loss at 36 weeks) than that in either the low burn (13% loss) or low mow (also 13% loss) plots. Since, as mentioned earlier, significant treatment effects on the rate of cellulose decomposition were found in these plots while nitrogen additions were still ongoing[11], the disappearance of such an effect in the year following habitat management suggests that the techniques employed were effective at reducing the earlier stimulation in decomposer activity.

**TABLE 2**
**Effects of Historical Nitrogen Addition and Recent Habitat Management on Soil Nitrogen Content**

| | Former N treatment (1989-1996) | |
|---|---|---|
| **Management** | **Control** | **High N** |
| Management burn | $26.0^{ab}$ | $30.6^{b}$ |
| High intensity mow | $22.8^{ab}$ | $24.7^{ab}$ |
| Simulated accidental burn | $15.2^{a}$ | $21.0^{ab}$ |
| Nitrogen: $F_{1,18} = 3.79$, $p > 0.05$ | | |
| Management: $F_{2,18} = 7.82$, $p < 0.001$ | | |
| Interaction: $F_{2,18} = 0.31$, $p > 0.05$ | | |

*Note:* All values given as g $m^{-2}$. Means with different letters are significantly different.

### *Above-Ground*

Prior to management, nitrogen addition resulted in a significant increase in shoot length, canopy density, and height[11]. Following management, a persistent, statistically significant effect of the earlier nitrogen additions was found for shoot length. In 2000, plants which received earlier nitrogen additions had 26% longer shoots than control plants ($F_{1,24} = 9.48$, $p < 0.05$), compared to a stimulation of 47% in 1996. However, no significant effect of management was seen (Fig. 3); historical nitrogen addition resulted in a persistent increase in shoot length, 5 years after additions ceased, of 31, 22, 10, and 37% for the low mow, high mow, management burn, and simulated accidental burn, respectively. Similar patterns were also seen for canopy height and density. These data suggest that it may take a considerable number of years before the legacy of nutrient enrichment disappears even with the use of more intensive management techniques.

Seedling invasion by *Deschampsia* following management was also significantly affected by both management type ($F_{3,24} = 7.94$, $p < 0.001$) and historical nitrogen addition ($F_{1,24} = 6.88$, $p < 0.05$), although unlike in the experiment receiving ongoing nitrogen additions (above), the interaction between these treatments was not significant in the recovery study. As in the ongoing nitrogen addition study, the highest number of grass seedlings were in the simulated accidental burn plots (5.5 in the control and 7.0 in the nitrogen treatment); *Deschampsia* establishment was significantly lower in the low mow plots (0.75 and 1.5 in control and nitrogen treatments respectively). Since even relatively low initial numbers of invading seedlings may result in a large increase

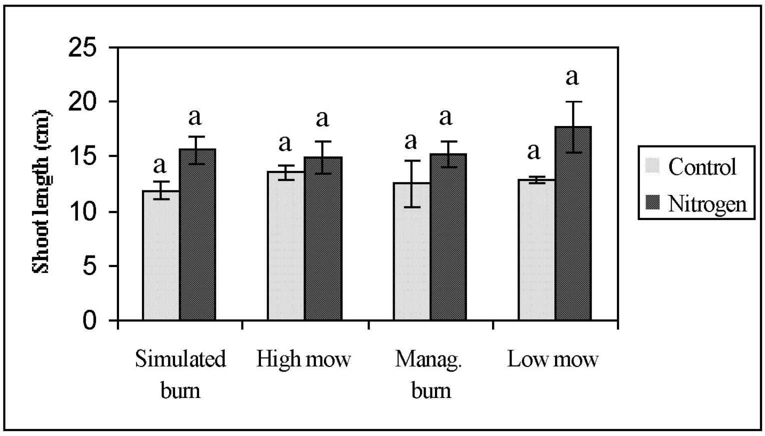

**FIGURE 3.** The effect of management and earlier (historical) nitrogen addition on shoot growth of *Calluna* in 2000. *Note:* Bars represent ±1 standard error; values with the same letters are not significantly different.

in grass seed inputs to the plots in subsequent seasons, small post-management differences may have important consequences for the species composition of plots in later years. The long-term persistence of invading species remains to be seen. Nevertheless, there is strong evidence from both nitrogen addition and recovery studies that nitrogen deposition promotes seedling invasion either directly or indirectly via accelerated nutrient cycling.

## Modelling Long-Term Responses

Although field experiments provide important insights into short-term ecosystem responses to nitrogen addition and the underlying mechanisms driving these responses, even the longest running ones represent only a small proportion of the life cycle of *Calluna.* Simulation models thus provide a tool with which to consider the long-term implications of nitrogen deposition for heathlands and the use of habitat management to mitigate deposition effects.

In The Netherlands, heather beetle outbreaks have been associated with gap formation in the *Calluna* canopy and are believed to represent the trigger for an increase in grass dominance at affected sites[32,33]. The occurrence of heather beetle outbreaks and the associated opening up of the *Calluna* canopy can therefore be used to indicate the point at which an opportunity for grass invasion is presented. Comparison between model runs with different simulated managements can thus provide an insight into the role of management in long-term community dynamics at heathland sites. As an illustration of the importance of habitat management, Fig. 4 shows model outputs under low-intensity and high-intensity mowing management at a

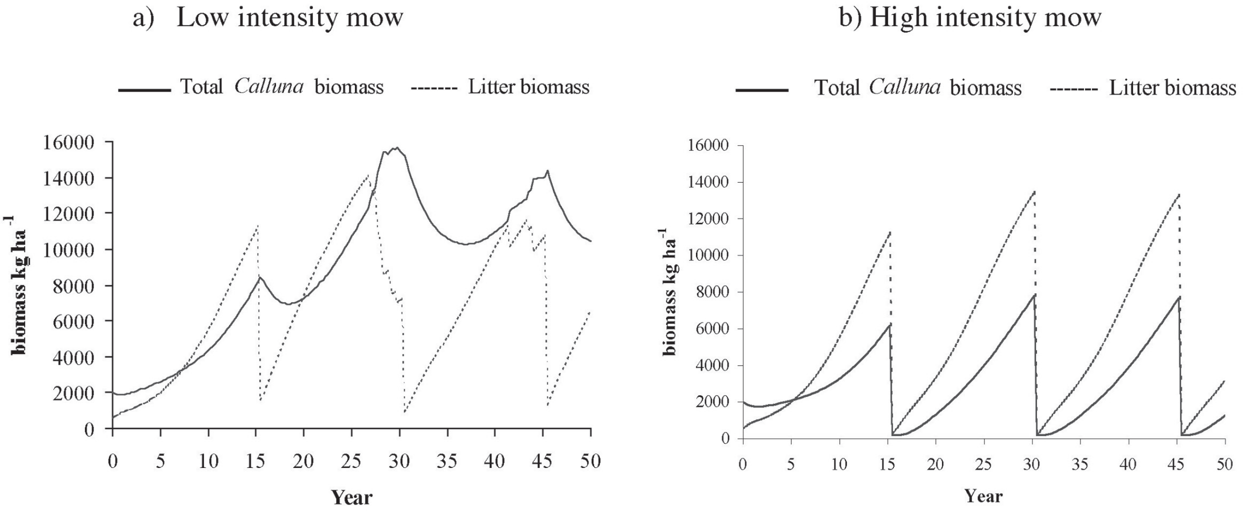

**FIGURE 4.** Model outputs for *Calluna* biomass from simulations at a deposition rate of 15 kg N ha$^{-1}$ year$^{-1}$ under (a) low-intensity and (b) high-intensity mowing managements. *Note:* CYG represents current year's growth.

deposition rate of 15 kg N $ha^{-1}$ $year^{-1}$ over three management cycles.

Heather beetle outbreaks are predicted to occur in year 26 under the low-intensity management, with a reduction in *Calluna* biomass occurring at this time. No outbreaks are predicted at lower deposition rates. This indicates that heathland managed using less intensive methods may be vulnerable to grass invasion in the gaps created by heather beetle damage in areas receiving deposition rates of 15 kg $ha^{-1}$ $year^{-1}$ or above. In contrast, when the model simulates a high-intensity mow with removal of the litter layer (Fig. 4b), heather beetle outbreaks are not predicted to occur below a deposition rate of 20 kg $ha^{-1}$ $year^{-1}$. The probability of heather beetle outbreaks in this model are controlled by litter biomass, foliar biomass, and foliar nitrogen concentrations, and it would be impossible to separate out the effects of these different factors experimentally. While this particular model only indicates an opportunity for grass invasion rather than simulating competition between dwarf shrubs and grasses, as has been illustrated in previous models[20], it nevertheless underlines the importance of habitat management in controlling the level of nutrients within the heathland and thus the ecosystem response to nitrogen deposition.

## CONCLUSIONS

The balance of evidence from both field experiments and model simulations suggests that more intensive managements which remove a significant proportion of vegetation and soil nitrogen pools may reduce the effect of nitrogen on heathland ecosystems relative to less intense managements. As such, management may provide a useful tool to mitigate, at least to some extent, the effects of atmospheric nitrogen deposition. However, it appears that even though management may promote heathland recovery from eutrophication, effects on vegetation are likely to persist for many years, especially in areas where atmospheric deposition remains high.

| | | |
|---|---|---|
| Received: | July | 28, 2001 |
| Revised: | November | 9, 2001 |
| Accepted: | November | 9, 2001 |
| Published: | December | 5, 2001 |
